# Engineering Excited States of Pt-Based Deep-Blue Phosphors to Enhance OLED Stability

**DOI:** 10.1021/acsomega.5c09501

**Published:** 2025-11-11

**Authors:** Yongjun Kim, Jaewook Kim, Woo Youn Kim

**Affiliations:** † Department of Chemistry, 34968 Korea Advanced Institute of Science and Technology, Daejeon 34141, Republic of Korea; ‡ Department of Supercomputing Acceleration Research, Division of National Supercomputing, 65351 Korea Institute of Science and Technology Information, Daejeon 34141, Republic of Korea

## Abstract

Platinum-based phosphorescent emitters have emerged as promising candidates for deep-blue OLEDs due to their high luminescence efficiency and narrow emission spectra. However, the insufficient chemical stability of blue phosphorescent emitters remains the primary obstacle to their commercial application. In this work, we present a systematic approach to enhance the stability of deep-blue emitters while preserving their optoelectronic properties. Using density functional theory calculations, we demonstrate that exciton-induced degradation via T_1_→^3^MC transition is the primary operational degradation pathway for *N*-heterocyclic carbene-based tetradentate Pt­(II) complexes. To suppress this pathway, we developed two complementary strategies that destabilize the ^3^MC state while maintaining T_1_ energy: (1) enhancing π-acceptance of the NHC moiety through electron-withdrawing substituents and (2) introducing steric hindrance via bulky groups on the pyridine ligand. Through systematic screening of PtON7 derivatives, we identified molecules with nearly doubled T_1_–^3^MC energy gaps compared to the parent compound, achieving considerable stabilization while maintaining deep-blue emission characteristics. These findings establish rational design principles for developing commercially viable blue phosphorescent OLEDs.

## Graphical abstract

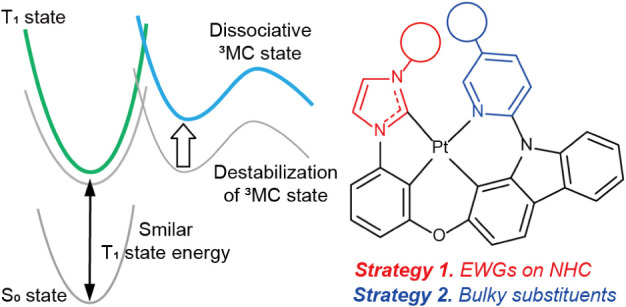

## Introduction

Organic light-emitting diodes (OLEDs) are highly successful devices in display and lighting technologies, offering numerous benefits, including reduced power consumption, high brightness, and flexible form factors.^1–3^ The development of transition metal-based phosphorescent emitters was a significant breakthrough that overcame the intrinsic efficiency limitations of purely fluorescent devices.^4^ Fluorescent emitters can only utilize singlet excitons, which account for 25% of the excitons generated electrically. In contrast, phosphorescent emitters utilize spin–orbit coupling to harvest both singlet and triplet excitons via intersystem crossing, allowing phosphorescent OLEDs (PhOLEDs) to approach 100% internal quantum efficiency.^5^ This fundamental advantage has led to the widespread commercialization of red and green PhOLEDs in display applications.^6–8^ However, blue PhOLEDs remain limited by insufficient operational stability, with even state-of-the-art devices achieving lifetimes at 95% of the initial value (LT_95_) below 300 h at 1000 cd/m^2^ ^9–16^ (Table S1)—far short of the industrial requirement of 5000 h.^17^ Consequently, the fluorescent blue emitters continue to be used in commercial applications to meet device lifetime standards. This stability-efficiency trade-off in blue OLEDs represents one of the most critical challenges facing the OLED industry.^18^

Among the various blue phosphorescent emitters, tetradentate platinum­(II) complexes have emerged as promising candidates.^19^ A well-known example is PtON7-dtb.^20^ Devices incorporating PtON7-dtb exhibit high photoluminescence quantum yields and narrow emission spectra, which are crucial for achieving both high efficiency and color purity in deep-blue OLEDs. The tetradentate coordination structure provides enhanced molecular rigidity compared to traditional bidentate Pt­(II) or Ir­(III) complexes because the ligand binds to the metal center with a rigid square-planar structure.^19^ As a result, these Pt emitters effectively suppress nonradiative decay and vibronic coupling, which improves the device efficiency and emission spectrum.^19^ Most tetradentate ligands incorporate controlled nonplanarity in their design. This intentional nonplanarity effectively prevents intermolecular Pt···Pt interactions and the aggregation-induced excimer formation, thereby preserving color purity.^21^

The incorporation of *N*-heterocyclic carbene (NHC) moieties has further advanced the design of tetradentate Pt­(II) emitters.^12,22–28^ The strong σ-donating ability of NHCs results in high-lying empty metal d-orbitals and strong metal–ligand bonds. This electronic modulation blue-shifts emission by increasing the HOMO–LUMO gap and enhances complex stability simultaneously.^29^ Recently developed blue emitters are taking advantage of this characteristic. In 2022, Sun and colleagues introduced PtON-TBBI (also known as BD-02), which demonstrated exceptional performance: an external quantum efficiency (EQE) exceeding 20%, a Commission Internationale de l’Eclairage y coordinate (CIE_*y*_) below 0.2, and an LT_95_ exceeding 100 h at an initial luminance of 1000 cd/m^2^.^9^ Subsequently reported devices utilizing PtON-tb-DTB and Pt2 emitters showed similar results, validating the effectiveness of the NHC-based design strategy.^10,14^ Despite these advances, the operational lifetime of blue PhOLEDs remains insufficient for commercial applications. All reported blue devices to date have achieved LT_95_ values below 300 h at 1000 cd/m^2^ (Table S1), far short of the industrial requirement.

The limited operational stability of blue OLEDs is usually attributed to the operational degradation of their constituent materials. The OLED device, especially the emissive layer, is subjected to various stress factors during operation, including charge carriers injected from electrodes and high-energy excitons generated through charge recombination.^30^ These species carry sufficient energy to break the covalent bonds of the constituent materials. In phosphorescent emitters, exciton-induced dissociation of the metal–ligand bond has been studied as the primary cause of device degradation. For both Ir­(III) and Pt­(II) complexes, the energy difference between the emissive triplet excited state (T_1_) and the metal–ligand dissociated state (known as the metal-centered state, ^3^MC) is usually small enough to be activated by thermal energy at room temperature.^31–33^ Researchers have been concentrating on elevating the energy of the ^3^MC state to improve the stability of blue phosphorescent emitters. In the case of Pt­(II) emitters, Kang and coworkers introduced intramolecular hydrogen bonding to increase the relative energy of the ^3^MC state, thereby creating a higher activation barrier for dissociation.^34^ However, modifying the structure of emitters changes their electronic structure, which can affect the emission color, efficiency, and other device characteristics. Therefore, we need to develop systematic strategies that can enhance molecular robustness without compromising optoelectronic performance.

In this work, we present a systematic approach to suppress exciton-induced degradation of NHC-based tetradentate Pt­(II) blue emitters while preserving their optoelectronic properties. We investigated the bond dissociation mechanisms of PtON7-dtb and related complexes using density functional theory (DFT) calculations, identifying Pt-pyridine bond cleavage via ^3^MC state formation as the primary degradation pathway. Through detailed electronic structure analysis, we developed two complementary design strategies: enhancing π-backdonation of NHC moiety to destabilize the ^3^MC state electronically, and introducing steric barriers to inhibit the T_1_→^3^MC transition geometrically. Our systematic screening of 75 substituents yielded six novel Pt­(II) complexes with nearly twice the T_1_–^3^MC energy gap of PtON7 while maintaining deep-blue emission characteristics. These findings establish rational design principles for developing commercially viable blue PhOLEDs.

## Methods

All calculations were performed using the Gaussian 16 software.^35^ The B3LYP density functional was employed for the calculations.^36^ For platinum atoms, the LANL2DZ basis set, along with its associated effective core potential, was employed.^37^ The 6–31G­(d) basis set was used for all other atoms.^38^ The T_1_ energy (Δ*E*_T1_) was obtained by calculating the adiabatic electronic energy difference between the optimized geometries of the lowest singlet (S_0_) and T_1_ states. All molecular orbital visualizations were performed using Chemcraft software (Version 1.8) with an isosurface value of 0.03 au^39^

π-backbonding interactions were analyzed via Natural Bond Orbital (NBO) analysis, following the methodology outlined in ref 40. Since π-backbonding refers to electron donation from metal d-orbitals to vacant ligand orbitals, we focused on identifying molecular orbitals with high Pt d-orbital character. NBO analysis confirmed that all four occupied lone pairs of Pt showed more than 90% d-orbital character. Therefore, π-backbonding energies were calculated by summing second-order perturbation theory energies (*E* ^(2)^) for all donor–acceptor interactions between Pt d-type lone pair orbitals (LP) and C–N π* orbitals of the NHC moieties as follows.

$$E_{\pi -\mathrm{backbonding}}^{\left(2\right)}=\underset{\pi^{*}\left(C-N\right),\mathrm{LP}\left(d\right)\mathrm{Pt}}{\sum }q_{\mathrm{LP}\left(d\right)\mathrm{Pt}}\frac{F{\left(\pi^{*}\left(C-N\right),\mathrm{LP}\left(d\right)\mathrm{Pt}\right)}^{2}}{\varepsilon_{\pi^{*}\left(C-N\right)}-\varepsilon_{\mathrm{LP}\left(d\right)\mathrm{Pt}}}$$

Here, *q*_LP*(d)*Pt_ is the orbital occupancy of the Pt d orbital, *ε*_π*(C–N)_,*ε*_LP(*d*)Pt_ are diagonal elements (orbital energies) of each orbital, and *F*(*π* ^*^(C–N),LP­(*d*)­Pt) is the off-diagonal NBO Fock matrix element.

## Result and Discussion

### Degradation Mechanism of PtON7-dtb

To develop design strategies for robust Pt-based blue emitters, we first investigated the exciton-induced degradation mechanisms of a model emitter molecule. We used PtON7-dtb as a representative example of tetradentate blue emitters, which suffers from insufficient operational stability despite its excellent photophysical properties: high EQE, narrow emission spectrum, and deep-blue color.^20^ As pointed out in our previous paper, multiple degradation pathways can contribute to emitter degradation.^41^ We began with a comprehensive analysis of bond dissociation pathways in the lowest triplet state (T_1_) of PtON7-dtb, then extended our investigation to related emitters sharing the same core structure (PtON7).^23^

Figure 1 presents the calculated Gibbs free energy profiles for single-bond dissociation reactions of PtON7-dtb. We systematically examined all single bonds in the core structure of the emitter, with each bond denoted by a different symbol in Figure 1a. C–H bond dissociation was excluded due to high dissociation energies. While ground-state C–H bond dissociation free energies (BDFEs) typically exceed 90 kcal/mol,^42^ these bonds remain strong even in the T_1_ excited state, requiring more than 40 kcal/mol for dissociation (except for benzylic hydrogens).^43^ Energy minima could not be found for Pt-benzene and Pt-carbazole bond dissociations (see energy scan results in the Figure S1). These bonds form a part of the rigid tetradentate chelating structure, making their dissociation geometrically unfavorable. Additionally, we calculated BDFEs in the triplet state for the complete detachment of functional groups, which includes the detachment of methyl and *tert*-butyl groups.

**Figure 1:**
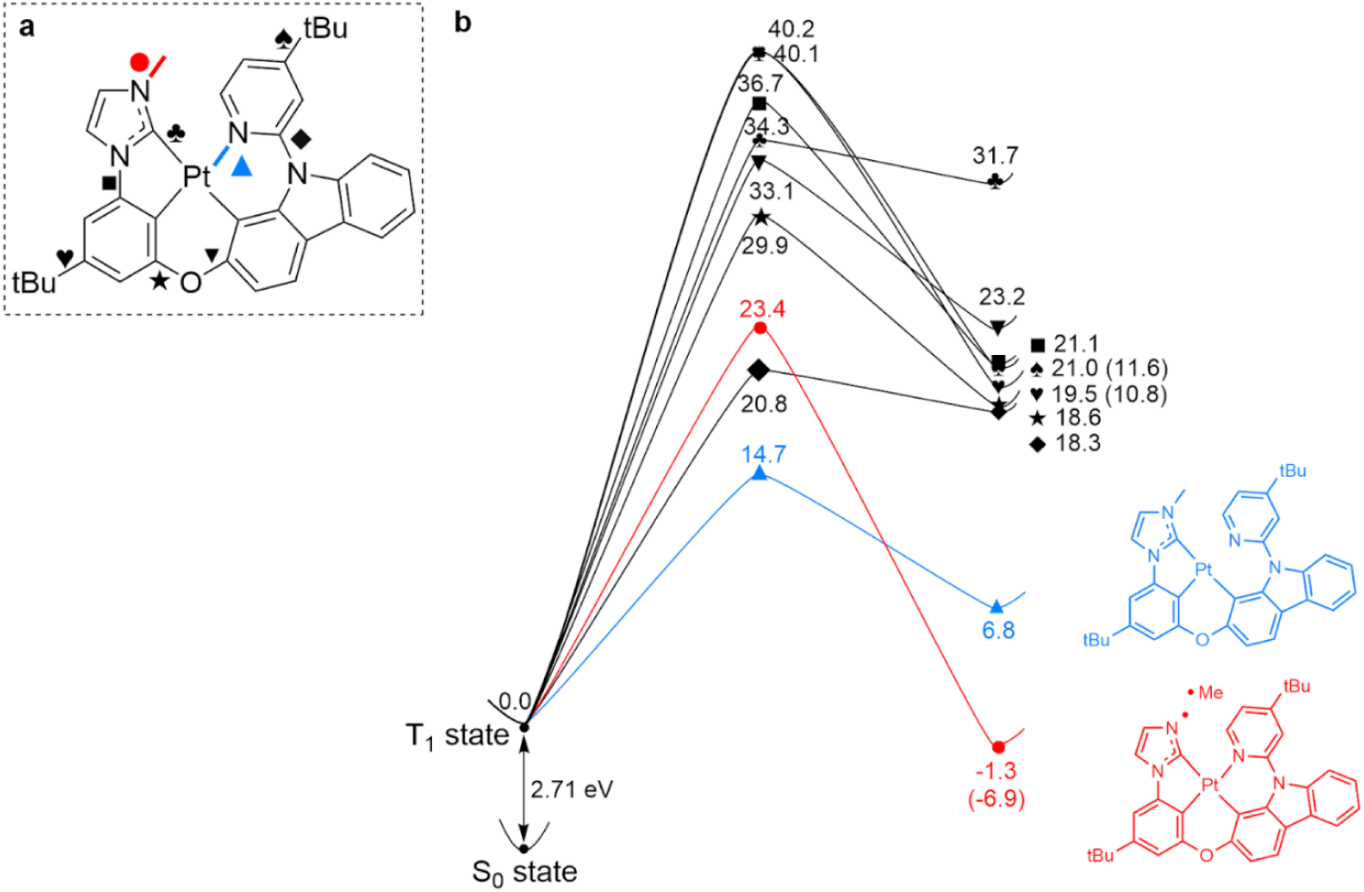
Energy profile of dissociation reaction of single bonds in the T_1_ excited state of PtON7-dtb (a) Structure of PtON7-dtb and examined single bonds. (b) Gibbs free energy profiles of bond dissociating reactions. All values are relative value to the Gibbs free energy of the T_1_ state. The values inside the parentheses are the excited-state bond dissociation Gibbs free energies. (Unit: kcal/mol).

Among the examined single bonds in the T_1_ state of PtON7-dtb, the Pt-pyridine bond (blue line, ▲) and the NHC-methyl bond (red line, ●) were identified as the most vulnerable to the dissociation reactions based on their kinetic and thermodynamic favorability, respectively, as shown in Figure 1b. The Pt-pyridine bond dissociation shows the lowest activation energy of 14.7 kcal/mol, making it the most kinetically favorable pathway. This dissociation forms a three-coordinate intermediate known as the metal-centered state (^3^MC). Furthermore, the ^3^MC structure is susceptible to further degradation, as the pyridine-carbazole bond (⧫) dissociation requires only 6.8 kcal/mol in terms of excited state BDFE. (Figure S2) In contrast, the NHC-methyl bond dissociation was identified as the only exothermic process, with the dissociated product being 1.3 kcal/mol more stable than the T_1_ state. Despite its high activation barrier of 23.4 kcal/mol, the NHC-methyl bond dissociation is the most thermodynamically favored pathway. These results suggest that both Pt-pyridine and NHC-methyl dissociation can be the dominant degradation pathways affecting the operational lifetime of blue OLED devices.

To determine which of these degradation pathways dominates device performance, we extended our analysis to a broader set of emitters. We collected operational lifetime data from the literature for 11 Pt­(II)-based blue emitters that share the same core structure as PtON7-dtb.^10–14^ We calculated the activation energies for both the Pt-pyridine and NHC-substituent bond dissociation pathways of each emitter molecule. In cases where the NHC bears substituents other than methyl groups (e.g., PtON-TBBI), we analyzed the corresponding NHC-substituent bond. Since device architecture significantly influences operational lifetime independent of emitter stability, we analyzed each device configuration separately to isolate the effect of emitter structure on degradation (see Table S1 for a complete list of emitters and their corresponding device data).

A positive correlation was observed between device lifetime and the activation energy for Pt-pyridine bond dissociation, as well as the energy gap between the T_1_ and ^3^MC states, across all device configurations (Tables S1, S2 and Figure S3). These results suggest that degradation pathways involving the ^3^MC state are the dominant mechanism underlying the instability of Pt-based blue emitters. The weaker correlation observed for the NHC-substituent dissociation, despite its thermodynamic favorability (negative excited-state BDFE values for all emitters studied), indicates that kinetic factors dominate under device operating conditions. This can be explained by the significant difference in activation barriers between the two pathways. The dissociation of NHC-substituent bonds in T_1_ state requires overcoming an energy barrier exceeding 20 kcal/mol for all investigated emitters. This barrier was 7–12 kcal/mol higher than the energy barrier for the dissociation of the Pt-pyridine bond. These findings support that the metal–ligand bond dissociation mechanism previously discussed in the introduction is the primary cause of exciton-induced degradation of Pt emitters.

While the above analysis focused on homolytic dissociation at the T_1_ state, heterolytic dissociation can also occur in the T_1_ state, and Pt emitters can exist at cation, and anion during device operation. To account for these possible degradation pathways, we explored dissociation pathways including heterolytic dissociation for the T_1_ state, cation, and anion. The calculation results showed that homolytic dissociation at the T_1_ state has lower activation energies, reaction energies, and BDFEs compared to all other dissociative pathways. (Tables S3–S6) While the NHC-Me bond at the anion showed similar BDFEs to the T_1_ state, the anion exhibited higher activation barriers. (Table S6) Moreover, for the 11 PtON7-based emitters with reported operational lifetimes, no correlation was found between anion NHC-Me BDFEs and device lifetime, even though several molecules exhibited weaker bonds in the anion state than in the T_1_ state (Table S2, and Figure S5). These findings confirm that homolytic dissociation in the T_1_ state is key to the operational lifetime of PtON7-based molecules.

Our analysis identifies the dissociation of Pt-pyridine at T_1_ state as the primary degradation pathway. However, other pathways, including NHC-substituent bond dissociation, may also contribute to device failure. The relatively low activation energies for several pathways suggest that multiple degradation channels could be operative even at room temperature. Based on these mechanistic insights, we propose molecular design strategies focused primarily on strengthening the Pt-pyridine bond and destabilizing the ^3^MC state. Additionally, we will explore introducing other substituents that increase the bond strength between the substituents and the PtON7 ligand core, thereby ensuring additional stability. This includes addressing the weak NHC-methyl bond in PtON7-dtb and the *tert*-butyl groups, which have excited-state BDFEs below 15 kcal/mol.

To facilitate the rapid screening of candidate molecules, we introduce Δ*G*_MC_ (the Gibbs free energy difference between the ^3^MC and T_1_ states) as a computationally efficient descriptor for Pt-pyridine bond stability. This descriptor correlates strongly with both activation energy (Δ*G* ^‡^_MC_, Pearson’s correlation coefficient, *R* = 0.78, see Figure S6) and device lifetime. Δ*G*_MC_ enables us to perform high-throughput molecular screening without computationally demanding transition state calculations.

### Geometric and Electronic Structure of the T_1_ and ^3^MC States of PtON7

To develop strategies that stabilize the Pt-pyridine bond while preserving photophysical properties, we must understand the electronic and geometric features of the degradation process. We therefore analyzed the PtON7 core structure, which represents the common scaffold of all tetradentate Pt­(II) NHC-based emitters examined. By studying this unsubstituted framework, we can identify how different molecular sites contribute to stability and emission characteristics.

Figure 2a shows the distribution of the highest occupied and lowest unoccupied molecular orbitals (HOMO and LUMO) of the ground state (S_0_) of PtON7. The HOMO is delocalized over the Pt d-orbital, benzene, and carbazole moiety, while the LUMO is predominantly localized on the pyridine moiety. The *N*-heterocyclic carbene (NHC) moiety showed negligible density in both HOMO and LUMO. Consequently, introducing substituents onto the NHC moiety should minimally perturb the Pt emitter’s electronic properties. We further analyzed the electronic structure of PtON7 in the T_1_ and ^3^MC states. As shown in Figure 2b, two singly occupied molecular orbitals (SOMOs) of the T_1_ state have similar shapes to the HOMO and LUMO of the S_0_ state. In the ^3^MC state, the low-energy SOMO resembles the HOMO of the S_0_ state. In contrast, the high-energy SOMO corresponds to a Pt–ligand σ* antibonding orbital with a dominant Pt d-orbital character. Therefore, the energy of the ^3^MC state is affected by the energy level of the metal–ligand antibonding orbitals.

**Figure 2:**
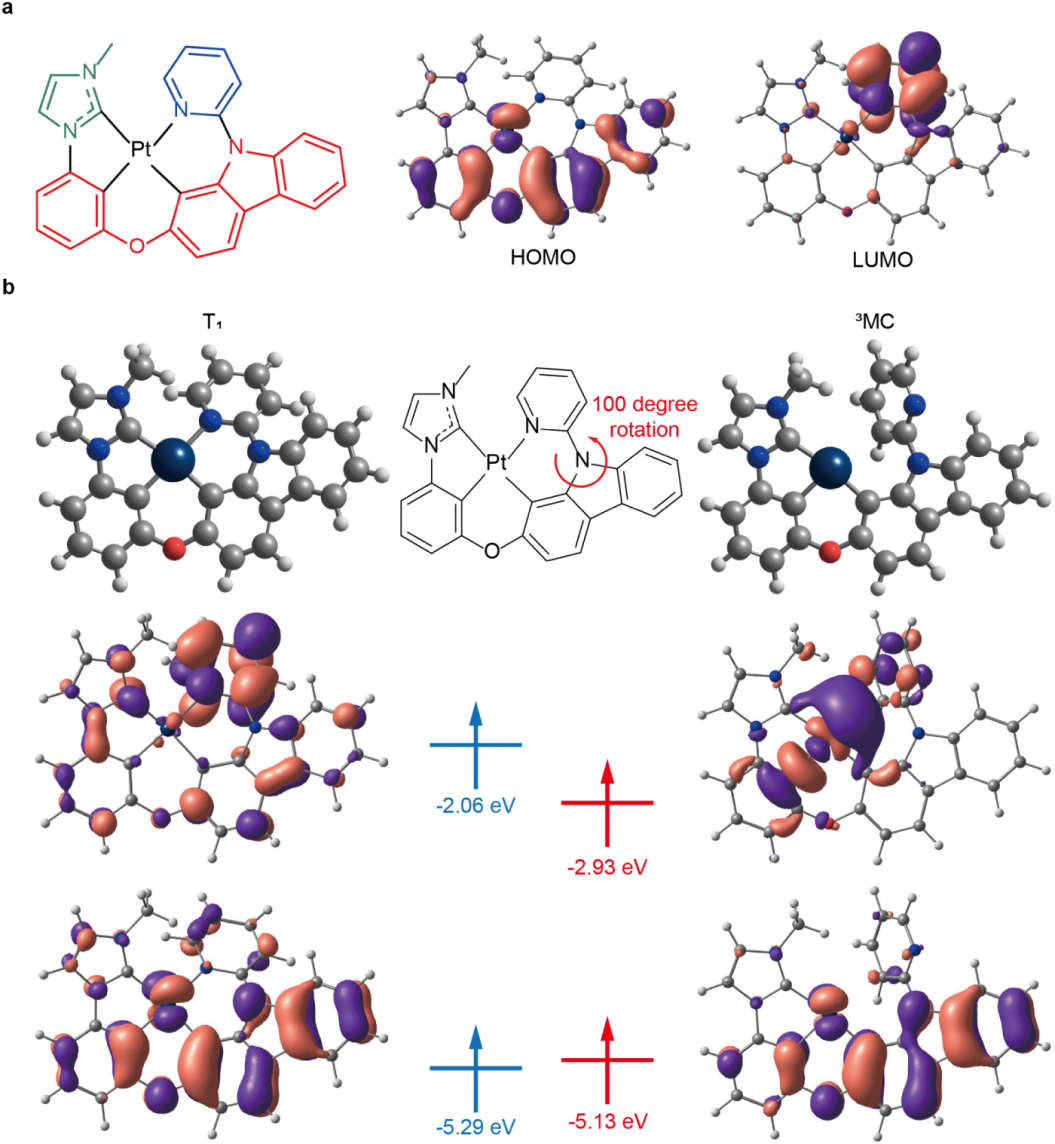
Electronic structure of PtON7. (a) Molecular structure, HOMO, and LUMO of PtON7 in S_0_ state. In molecular structure, the moieties where HOMO is localized are colored red, the moieties where LUMO is localized are colored blue, and the auxiliary ligand moiety is colored green. (b) Frontier orbitals and their energies in the T_1_ and ^3^MC state of PtON7.

We also analyzed the geometric difference between T_1_ and ^3^MC states (Figure 2b). The transition from the T_1_ to the ^3^MC state involves a significant geometric rearrangement, including an approximately 100° rotation of the pyridine moiety around the pyridine-carbazole bond axis. This geometric change suggests that introducing bulky substituents on either the pyridine or NHC moieties can induce steric barriers, leading to an increased activation energy for the T_1_ to ^3^MC transition.

Based on the above analysis, we propose two strategies to increase Δ*G*_MC_ while maintaining similar electronic properties. The first strategy is raising the energy of the Pt–ligand σ* antibonding orbital. To achieve this, we introduced various substituents onto the NHC moiety, modulating the Pt–ligand antibonding orbital energy and increasing the energy of the ^3^MC state. The second strategy is introducing bulky substituents onto the pyridine ligand. These bulky substituents result in steric hindrance between the pyridine and the adjacent NHC moiety, destabilizing the ^3^MC state.

### Strategy for Increasing the Energy Level of the Pt-Ligand Anti-Bonding Orbital

To control the electronic structure of Pt emitters by introducing substituents onto the NHC moiety, we first examined the electronic properties of NHC. The NHC exhibits strong metal binding due to its σ-donor and π-acceptor characters. The lone pair at the sp^2^-hybridized orbital of the carbene center donates electrons to the metal (σ donation), and the unoccupied carbon 2p orbital accepts electron density from the metal d orbitals (π-backdonation).^44^ As the NHC accepts more electrons from the metal via π-backdonation, the π-acceptor character of NHC becomes stronger, destabilizing the metal–ligand σ* antibonding orbital and elevating its energy (Figure 3a).^45^

**Figure 3:**
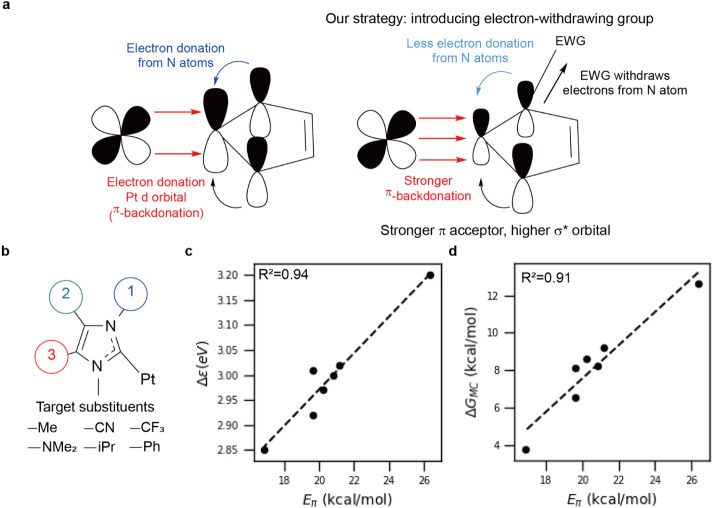
Introducing electron-withdrawing groups to destabilize the ^3^MC state of PtON7. (a) Scheme of our strategy to increase Δ*G*_MC_ by introducing electron-withdrawing groups. (b) The type and position of the introduced substituents. (c) Correlation between *E*_π_ and the energy gap between the high-energy SOMO and σ* orbital (Δε) at T_1_ state. (d) Correlation between the π-backbonding energy and Δ*G*_MC_.

Motivated by the unique electronic characteristics of NHC, we aimed to raise the σ* orbital energy by enhancing the π-backdonation, thereby increasing Δ*G*_MC_. We hypothesized that electron-withdrawing groups (EWGs) on the NHC moiety make the carbene center more electron-deficient, increasing π-backdonation from the Pt d-orbital. This enhanced π-backdonation shortens the Pt-NHC bond distance, leading to increased overlap between the NHC lone pair and Pt d-orbital. This increased orbital overlap strengthens the σ-bonding interaction, leading to increasing the σ* orbital energy and so widening the σ-σ* energy gap. Since the ^3^MC state involves electron population of this σ* orbital, higher σ* energy destabilizes the ^3^MC state. This destabilization renders the T_1_ → ^3^MC transition energetically unfavorable, thereby suppressing the Pt-ligand bond dissociation. (Figure 3a) To validate this hypothesis, we systematically investigated how both electron-withdrawing and electron-donating substituents affect ΔG_MC_ by introducing various small functional groups such as CN and CF_3_ (EWGs), Me and Ph (neutral), and NMe_2_ and iPr (electron donating groups) at three different positions on the NHC moiety (Figure 3b and Table S7). Note that synthetic accessibility was not considered at this stage. In this section, our goal is to validate the hypothesis through computational experiments designed to isolate purely electronic effects on π-backdonation and Pt-pyridine bond strength.

DFT calculations confirmed that the electronic nature of the substituent on the NHC moiety can modulate Δ*G*_MC_, with the effect varying by the substitution position. Natural Bond Orbital (NBO) analysis quantified the π-backdonation strength (*E*_π_) for each substituted complex. As shown in Table 1, *E*_π_ varies up to 7 kcal/mol for substituents at position 1, while positions 2 and 3 show minimal variation (±2.3 kcal/mol). This positional selectivity arises because substituents at position 1 directly influence the electron density at the nitrogen atom adjacent to the carbene center, thereby modulating the carbene’s π-acceptor capability. Specifically, at position 1, EWG substituents such as CN and CF_3_ exhibited higher *E*_π_ values than that of Me, while iPr, which is known as an electron-donating group, showed a lower *E*_π_ value.

**Table 1:** Relative π-Backdonation Strengths (Δ*E*_π_)­^a^ of Substituted Derivatives

Δ*E*_π_ (kcal/mol)	Me	CN	CF_3_	NMe_2_	iPr	Ph
Position 1	0.0 (PtON7)	6.8	1.2	0.6	–2.7	0.0
Position 2	–0.3	2.0	1.2	–0.2	–0.5	0.1
Position 3	1.0	2.4	2.6	0.6	1.5	1.0

a Δ*E*_π_ values are given relative to PtON7 (Δ*E*_π_ = 19.7 kcal/mol).

To confirm our proposed hypothesis, enhancing π-backbonding will increase Δ*G*_MC_ by elevating the σ* orbital energy, we examined the effect of π-backbonding on the σ* orbital energies. We focused our analysis on molecules with substituents at position 1, as this site exhibited the most significant variation in *E*_π_ among all possible substitution positions. The electronic structures of all molecules are presented in the Table S7. To investigate how the π-backdonation affects the σ* orbital energy, we examined the relationship between *E*_π_ and the energy gap between the high-energy SOMO and σ* orbital in the T_1_ state (Δε). A linear correlation was observed between Δε and *E*_π_, indicating that stronger π-backdonation elevates the σ* orbital energy (Figure 3c). This elevation in the σ* orbital energy contributes to the increase in Δ*G*_MC_, as confirmed by the linear correlation between *E*_π_ and Δ*G*_MC_ (Figure 3d). Therefore, introducing EWGs at position 1 of the NHC moiety provides a rational design principle to stabilize Pt emitters by strengthening the vulnerable Pt-pyridine bond.

### Strategy for Destabilizing the ^3^MC State Through Steric Hindrance

Our second strategy focuses on destabilizing the ^3^MC state through steric repulsion. Since the T_1_ to ^3^MC transition of PtON7-like emitters causes significant pyridine rotation, introducing bulky substituents on the pyridine moiety increases steric hindrance with the NHC moiety in the ^3^MC state. Since the LUMO is predominantly localized on the pyridine moiety, we selected alkyl groups as substituents due to their minimal electronic perturbation while providing the desired steric bulk. As shown in Figure 4a, the pyridine ring offers four possible sites for substitution. However, position 4 was excluded from the candidates due to the high steric hindrance between the substituent and the NHC moiety, even in the ground-state geometry. We systematically introduced five substituents (methyl (Me), tert-butyl (tBu), adamantane (Ada), phenyladamanane (PhAda), and mesitylene (Mes)) at positions 5, 6, and 7 to evaluate changes in Δ*G*_MC_ (Figure 4b).

**Figure 4:**
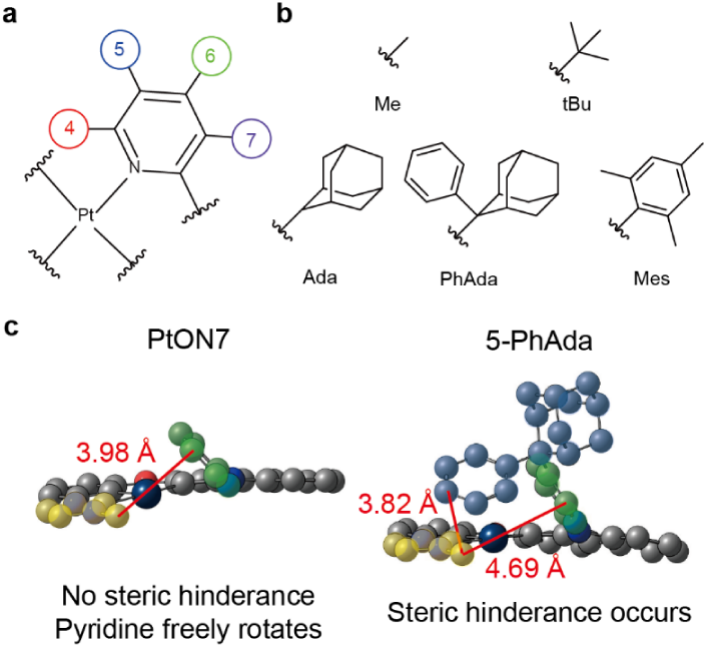
Introducing bulky substituents to destabilize the ^3^MC state. (a) Possible substitution positions and (b) Types of introduced substituents. (c) ^3^MC state structure of PtON7 and 5-PhAda. Pyridine, NHC, and PhAda moieties are colored green, yellow, and blue, respectively. Hydrogens are omitted for visual clarity.

DFT calculations showed that bulky substituents (PhAda and Mes) at positions 5 and 6 increased Δ*G*_MC_, while substituents at position 7 decreased Δ*G*_MC_ (Table 2). During the T_1_ to ^3^MC transition, the pyridine rotation brings the bulky alkyl group into proximity with the NHC moiety and its substituent (Me, in PtON7) and exhibits strong steric hindrance with NHC. Figure 4c shows that the PhAda substituent in position 5 (5-PhAda) extends perpendicular to the pyridine plane, inducing strong steric repulsion with the methyl group of the NHC moiety in the ^3^MC state. The Mes substituent shows a similar steric effect. Position 7 proved unsuitable as substituents at this site cause steric repulsion with the carbazole moiety even in the T_1_ state, weakening the Pt-pyridine bond. Interestingly, 6-Ada has a smaller Δ*G*_MC_ than PtON7 despite the Ada group being bulky enough. This is because the Ada group in position 6 increases the LUMO energy (see Table S8 for the orbital energies of the examined molecules), thereby elevating the energy of the T_1_ state.

**Table 2:** Effect of Bulky Substituents on T_1_ and ^3^MC State Energies

Molecule	PtON7	5-Me	5-tBu	5-Ada	5-PhAda	5-Mes
ΔΔ*E*_T1_ ^a^	0.00	0.00	0.04	0.02	0.00	–0.01
ΔΔ*G*_MC_ ^b^	0.00	3.73	4.33	3.83	4.93	6.43
BDFE^c^		32.2	13.5	17.3	–8.5	33.4

Molecule		6-Me	6-tBu	6-Ada	6-PhAda	6-Mes
ΔΔ*E*_T1_ ^a^		0.05	0.04	0.10	0.00	–0.03
ΔΔ*G*_MC_ ^b^		3.33	3.73	2.03	5.93	4.73
BDFE^c^		29.9	11.5	13.8	–9.3	30.1

Molecule		7-Me	7-tBu	7-Ada	7-PhAda	7-Mes
ΔΔ*E*_T1_ ^a^		–0.03	–0.05	–0.04	–0.10	–0.06
ΔΔ*G*_MC_ ^b^		–0.47	–4.67	–2.47	–2.17	3.93
BDFE^c^		21.7	–7.8	1.8	–34.4	19.2

a ΔΔ*E*_T1_ represents the variation in T_1_ excitation energy, Δ*E*_T1_, relative to the reference structure PtON7 (Δ*E*_T1, PtON7_ = 2.67 eV). (Unit: eV)

b ΔΔ*G*_MC_ represents the variation in the energy gap between ^3^MC and T_1_ state, Δ*G*_MC_, relative to the reference structure PtON7 (Δ*G*_MC, PtON7_ = 6.6 kcal/mol). (Unit: kcal/mol)

c BDFE refers to the bond dissociation free energy of the pyridine-substituent bond in T_1_ state. (Unit: kcal/mol)

To make our emitter design strategy effective, it is essential to evaluate potential degradation pathways arising from the introduced substituents. For example, in PtON7-dtb, the T_1_ state BDFEs of the *tert*-butyl groups, approximately 12 kcal/mol, are lower than the Pt-pyridine bond, suggesting the possibility of an alternative degradation pathway. We compared the excited-state BDFE of the pyridine-substituent bond for each molecule to verify that they exceed the Pt-pyridine bond strength (Table 2). The BDFE values ranged from −34 to 33 kcal/mol, showing strong dependence on both substituent type and position. Position 5 consistently showed the highest BDFEs compared to positions 6 and 7. Most substituents at positions 5 and 6 exhibited a higher BDFE than the Δ*G*_MC_ value, confirming that the pyridine-substituent bond is more stable than the Pt-pyridine bond. A notable exception is PhAda, which showed negative BDFE values at all positions. This likely results from the stability of the benzylic radical formed upon dissociation. Based on the combined criteria of maximizing Δ*G*_MC_ while maintaining a high BDFE, the 5-Mes is optimal, yielding Δ*G*_MC_ = 9.1 kcal/mol with a BDFE of 33.4 kcal/mol.

### Design of Stable Pt-Based Emitter Molecules

We proposed two strategies for developing stable Pt-based emitters. The first strategy is introducing EWGs into NHC to destabilize the ^3^MC state by raising the σ* orbital level. The second strategy is introducing bulky substituents into pyridine to destabilize the ^3^MC state through increased steric hindrance. Having validated both strategies independently, we combined them to maximize stability improvements. The electronic (EWG on NHC) and steric (bulky groups on pyridine) approaches operate through orthogonal mechanisms, suggesting their effects should be additive. We performed a two-step screening process to identify optimal candidates that maintain the photophysical properties of PtON7 while enhancing stability.

In the first step, we investigated substituents for NHC to select optimal substituents. We adopted various electron-withdrawing substituents, which can be categorized into four different types: (1) acceptor moieties from thermally activated delayed fluorescence (TADF) and Ir/Pt-based emitters, (2) Ph groups substituted with EWG, (3) classical EWGs, and (4) heterocyclic rings from PubChemQC.^11,46–49^ The heterocyclic rings were obtained from the PubChemQC database due to their usage as electron acceptor moieties in TADF and phosphorescence OLEDs. Figure S7 shows a complete list of the 75 substituents used in this paper. We considered two criteria for screening: (1) maintaining the emitter’s Δ*E*_T1_ and (2) achieving higher Δ*G*_MC_ than PtON7. Since PtON7 exhibits a Δ*E*_T1_ of 2.67 eV, molecules exhibiting Δ*E*_T1_ > 2.6 eV were selected to maintain similar T_1_ energy.

In the second step, we introduced a Mes group at position 5 for molecules that met all screening criteria in the first step. Subsequently, stricter criteria were applied to the molecules, with PtON-TBBI serving as the benchmark. This compound, with Δ*G*_MC_ = 9.2 kcal/mol, is widely used as a reference emitter in recent studies of Pt-based blue OLED lifetimes. Our final selection required: (1) Δ*G*_MC_ > 9.2 kcal/mol, (2) Δ*E*_T1_ > 2.6 eV to maintain blue emission, (3) HOMO/LUMO energy levels within ± 0.1 eV of PtON-TBBI to ensure compatibility with existing device architecture, and (4) both Δ*G* ^‡^_MC_ and BDFE of the NHC-substituent bond in T_1_ state (Δ*G*_NHC‑sub_) exceeding PtON-TBBI values.

Figure 5a illustrates the distribution of candidates in the Δ*G*_MC_–Δ*G*_NHC‑sub_ space, with the gray lines indicating PtON-TBBI reference values. Molecules in the upper-right quadrant surpass PtON-TBBI in both metrics. From these, six molecules (Figure 5b) met all criteria while retaining electronic structures suitable for deep-blue emission, as well as HOMO and LUMO orbital characteristics consistent with PtON7 (Table 3, Figure S8). All six designed molecules showed substantial improvements over the parent PtON7 (Δ*G*_MC_ = 6.6 kcal/mol). PtON-Mes-N1, N2, and N3 achieved Δ*G*_MC_ values of 11.7, 11.0, and 10.8 kcal/mol, respectively, with Δ*G* ^‡^_MC_ values comparable to state-of-the-art emitters. The fluorinated derivatives PtON-Mes-F1 and F2 demonstrated even higher ΔG^‡^_MC_ and Δ*G*_MC_ values compared to the PtON7-dtb and PtON7-TBBI. To verify their stability, we further examined the single bonds in fluorinated substituents. We calculated the BDFEs in triplet excited state, and all C–F bonds in PtON7-Mes-F1 and F2 exceeded 56 kcal/mol (Figure S9), supporting the stability of these candidates. For reference, ground-state benzene-fluorine BDFE is known as 127 kcal/mol, which is higher than C–C bond^50^ PtON-Mes-O1 exhibited the highest Δ*G*_MC_ of 12.0 kcal/mol, nearly doubling the stability metric of PtON7. However, the furan moiety may be susceptible to degradation by oxygen or UV light.^51^ and requires further stability assessment under device conditions. Importantly, these improvements were achieved while maintaining emission wavelengths and frontier orbital energies within the target range for deep-blue emission (Table 3). The results show that our strategies, which carefully adjust the electronic and geometric structure of the Pt­(II) emitter, successfully suppress the exciton-induced degradation of the Pt-pyridine bond while preserving their photoelectronic properties.

**Figure 5:**
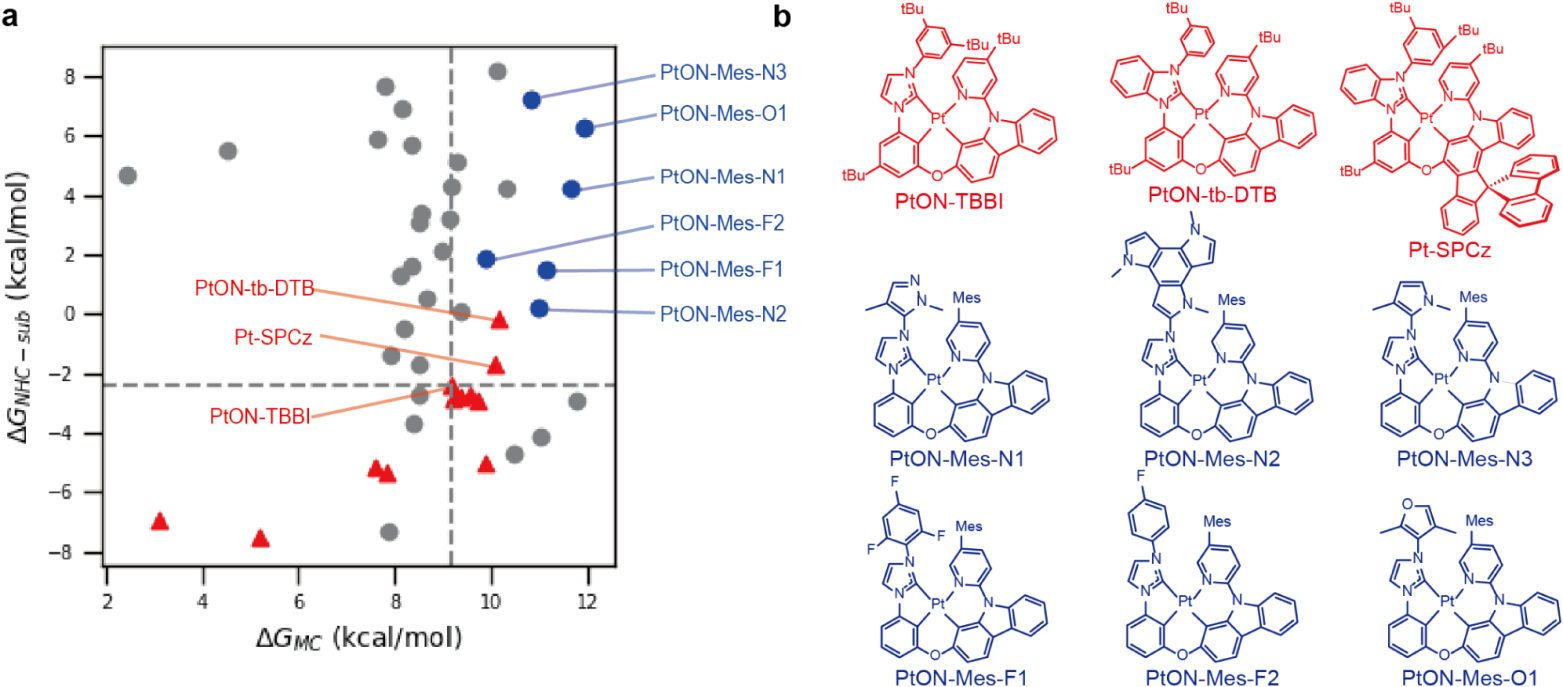
Structure of Pt emitters designed with two strategies. (a) Distribution of Δ*G*_MC_ and Δ*G*_NHC‑sub_ of candidate molecules. (Gray and blue dots) The gray vertical and horizontal dashed lines represent the values of Δ*G*_MC_ and Δ*G*_NHC‑sub_ for PtON-TBBI, respectively. Red dots indicate reported emitters. (b) The structure of previously reported emitters (shown in red) and robust blue Pt­(II) emitter candidates proposed in this paper (shown in blue), which are marked as blue dots in panel (a).

**Table 3:** Electronic Structure and Reaction Energy Profiles of the Well-Known Blue Pt­(II) Emitter and Robust Blue Pt­(II) Emitter Candidates

	Δ*E*_T1_ (eV)	HOMO (eV)	LUMO (eV)	Δ*G* ^‡^_MC_ (kcal/mol)	Δ*G*_MC_ (kcal/mol)	Δ*G*_NHC‑sub_ (kcal/mol)
PtON7	2.67	–4.67	–1.33	14.8	6.6	–5.8
PtON7-dtb	2.71	–4.58	–1.20	14.7	6.8	–6.9
PtON-TBBI	2.67	–4.64	–1.21	14.6	9.2	–2.4
PtON-Mes-N1	2.70	–4.76	–1.28	14.4	11.7	4.2
PtON-Mes-N2	2.71	–4.62	–1.15	13.8	11.0	0.2
PtON-Mes-N3	2.72	–4.69	–1.22	13.7	10.8	7.2
PtON-Mes-F1	2.65	–4.68	–1.30	15.4	11.1	1.5
PtON-Mes-F2	2.68	–4.69	–1.27	15.1	9.9	1.9
PtON-Mes-O1	2.69	–4.66	–1.24	13.9	12.0	6.3

## Conclusion

In this paper, we propose a systematic approach to improve the operational stability of Pt-based emitters. By analyzing energy profiles of various bond dissociation pathways of PtON7-dtb, we identified that the dissociation of the Pt-pyridine bond is correlated with the lifetime of Pt OLED devices. Detailed electronic structure analysis showed that the dissociation of the Pt-pyridine bond can be effectively suppressed with two independent approaches: (1) introducing electron-withdrawing groups on the NHC moiety to elevate the energy of the Pt–ligand antibonding orbital through enhanced π-backdonation, and (2) incorporating sterically bulky substituents on the pyridine ring to destabilize ^3^MC state via steric hindrance with the NHC moiety. By combining these two strategies, we designed new Pt-based emitters with stronger Pt-pyridine and NHC-substituent bonds. The designed emitters, particularly PtON-Mes-N1 and PtON-Mes-N2, achieve ΔG_MC_ values up to 11.7 kcal/mol, which is nearly double that of the parent PtON7 (6.6 kcal/mol), while maintaining the crucial optoelectronic properties. These results suggest that the two strategies can improve the stability of Pt emitters while maintaining their optical characteristics. Therefore, we anticipate that our design strategies will contribute to the development of robust Pt-based phosphorescent emitters.

## Supplementary Material



## Notes

Potential energy surface scans for bond dissociation pathways of PtON7-dtb; correlation analysis between calculated energetic parameters (Δ*G* ^‡^_MC_, Δ*G*_MC_, Δ*G* ^‡^_NHC‑sub_, Δ*G*_NHC‑sub_) and experimental device lifetimes; comprehensive survey of operational lifetimes for Pt­(II)-based blue OLEDs; detailed energetic parameters for literature-reported Pt­(II) emitters; list of 75 electron-withdrawing substituents screened for NHC modification; electronic structure data for all substituted PtON7 derivatives; C–F bond dissociation energy of PtON7-Mes-F1 and PtON7-Mes-F2 (PDF)
