# Supplementary material for: Engineering Excited States of Pt-Based Deep-Blue Phosphors to Enhance OLED Stability
Source: ACS Omega. 2025 Nov 11;10(46):56753–62. doi: 10.1021/acsomega.5c09501 (PMC12658782; doi:10.1021/acsomega.5c09501)
Supplement: Supplementary file 1 [file ao5c09501_si_001.pdf]

Supporting Information

of

Engineering Excited States of Pt-based Deep-Blue Phosphors to Enhance OLED Stability

Yongjun Kim[a], Jaewook Kim[b*], and Woo Youn Kim[a*]

a) Department of Chemistry, KAIST, Daejeon 34141, Republic of Korea

b) Department of Supercomputing Acceleration Research,
Division of National Supercomputing,
Korea Institute of Science and Technology Information,
Daejeon 34141, Republic of Korea

*E-mail: jaewookkim@kisti.re.kr;

*E-mail: wooyoun@kaist.ac.kr

Contents

Figure S1. Potential energy surfaces of PtON7-dtb along the elongation of the Pt–benzene and Pt–pyridine bonds.

Figure S2. Gibbs free energy profiles for dissociation of the Pt-pyridine bond (blue line) and the Pt-NHC bond (red line). All values are reported relative to the T_1 state energy.

Figure S3. Correlation between calculated energetic parameters ($\Delta G^{\ddagger}_{MC}$, ΔG_{MC}, $\Delta G^{\ddagger}_{NHC-sub}$, and $\Delta G_{NHC-sub}$) of previously reported PtON7-based emitters and their experimental OLED device lifetime.

Figure S4. Potential energy surfaces of PtON7-dtb along the elongation of the benzene-O, and Carbazole–O bonds

Figure S5. Correlation between $\Delta G_{NHC-sub}$ of previously reported PtON7-based emitters and their experimental OLED device lifetime.

Figure S6. Correlation between $\Delta G^{\ddagger}_{MC}$ and ΔG_{MC} of previously reported PtON7-based emitters. The complete list of emitters is provided in Table S3.

Figure S7. Chemical structures of substituents investigated for the NHC moiety to enhance Pt-pyridine bond stability.

Figure S8. Frontier orbitals of PtON-Mes molecules.

Figure S9. C-F bond dissociation free energies of PtON7-Mes-F1 and PtON7-Mes-F2 in triplet excited state

Table S1. Operational lifetimes and device structures of previously reported Pt-based blue OLED emitters.

Table S2. $\Delta G^{\ddagger}_{MC}$, ΔG_{MC}, $\Delta G^{\ddagger}_{NHC-sub}$, and $\Delta G_{NHC-sub}$ of previously reported Pt OLED molecules.

Table S3. Bond dissociation free energies in the T_1 state for the complete detachment of functional groups (R)

Table S4. Bond dissociation free energies in the cationic state for the complete detachment of functional groups (R)

Table S5. Bond dissociation free energies in the anionic state for the complete detachment of functional groups (R)

Table S6. Energetics of bond dissociation pathways for PtON7-dtb in charged states. (Unit: kcal/mol)

Table S7. Electronic structures of PtON7 derivatives according to substituent types and positions on the N-heterocyclic carbene ring.

Table S8. Electronic structure PtON7 derivatives according to substituent types and positions on the pyridine ring

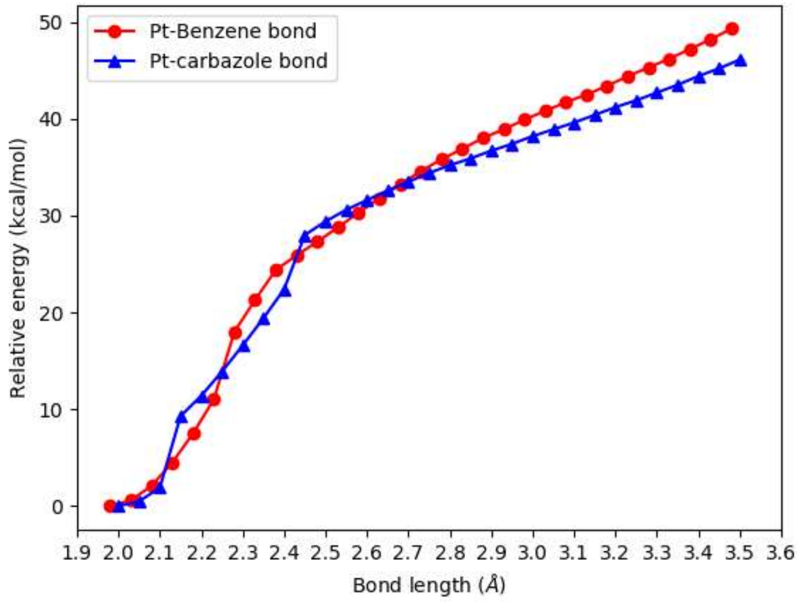

Figure S1. Potential energy surfaces of PtON7-dtb along the elongation of the Pt–benzene and Pt–pyridine bonds.

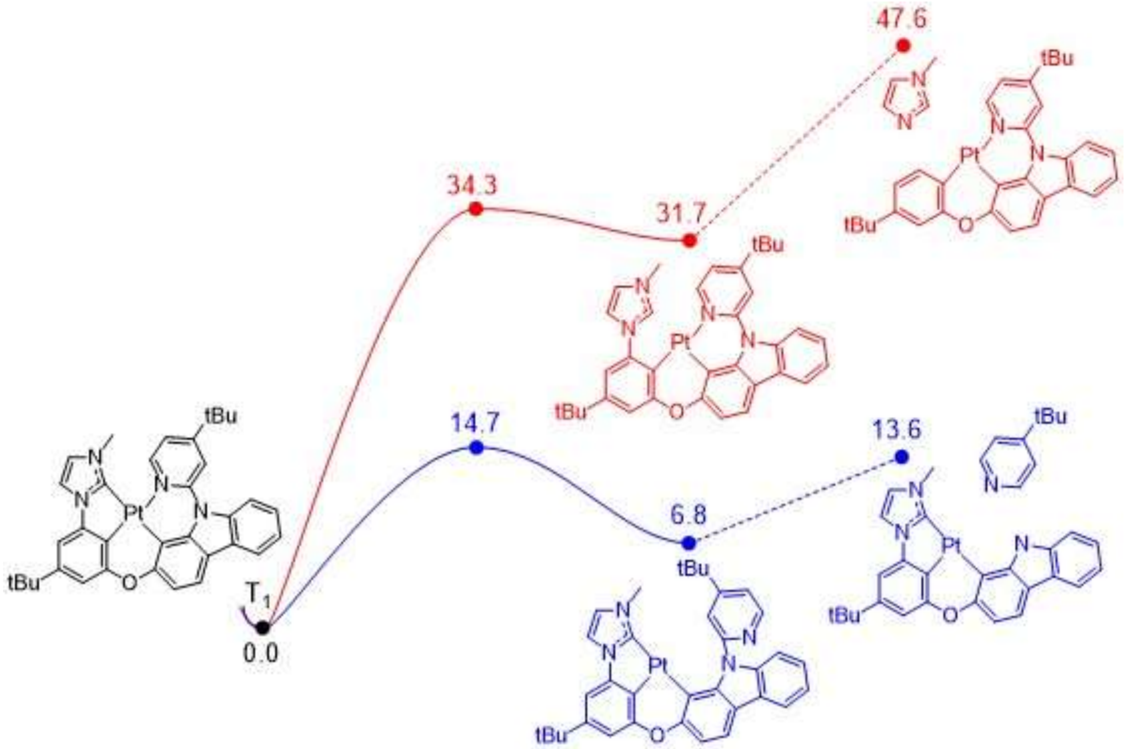

Figure S2. Gibbs free energy profiles for dissociation of the Pt-pyridine bond (blue line) and the Pt-NHC bond (red line). All values are reported relative to the T_1 state energy. (Unit: kcal/mol)

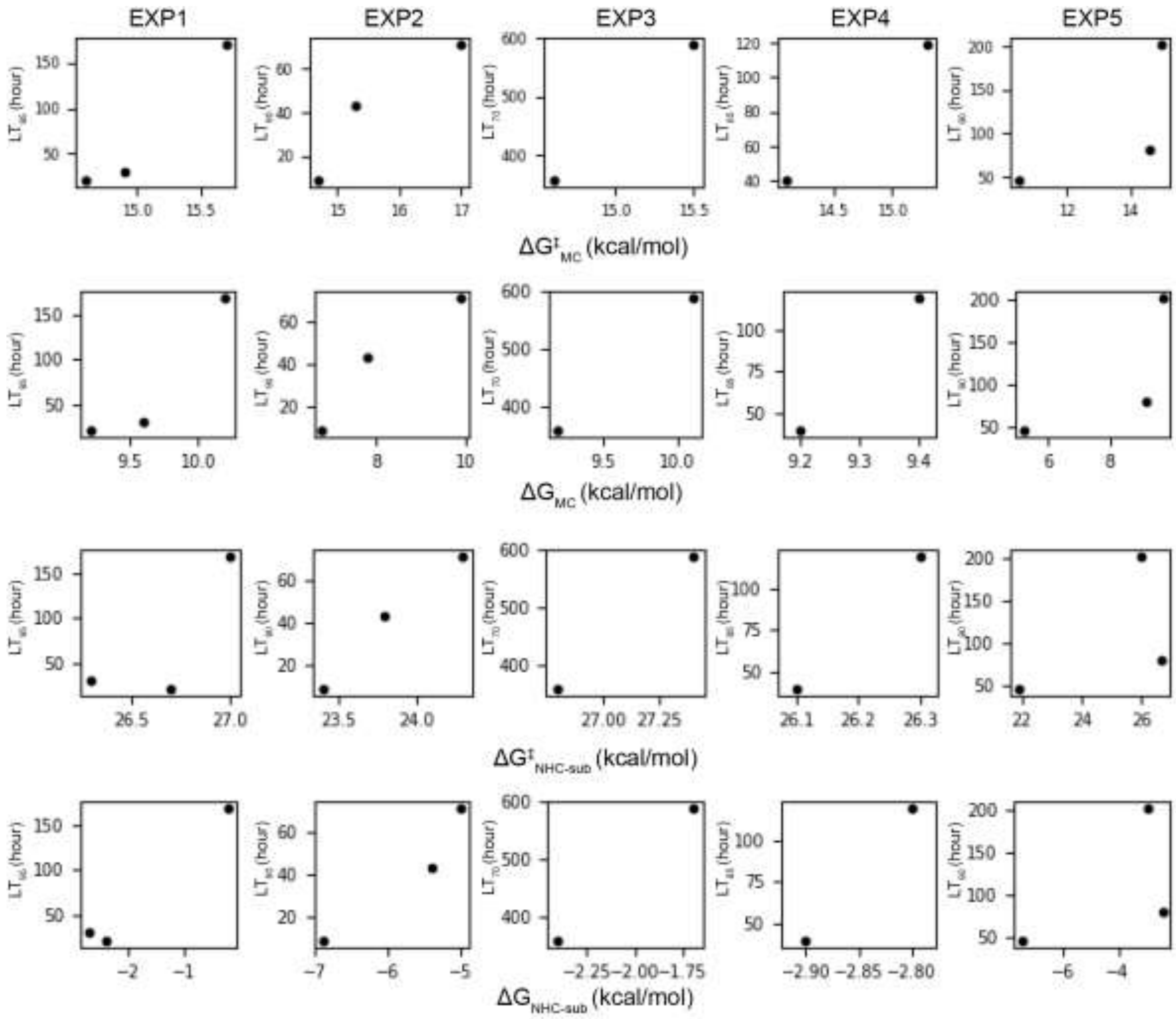

Figure S3. Correlation between calculated energetic parameters ($\Delta G^{\ddagger}_{MC}$, ΔG_{MC}, $\Delta G^{\ddagger}_{NHC\text{-}sub}$, and $\Delta G_{NHC\text{-}sub}$) of previously reported PtON7-based emitters and their experimental OLED device lifetime. The details of each device are summarized in Table S1. (Units: energetic parameters in kcal/mol; lifetimes in hours)

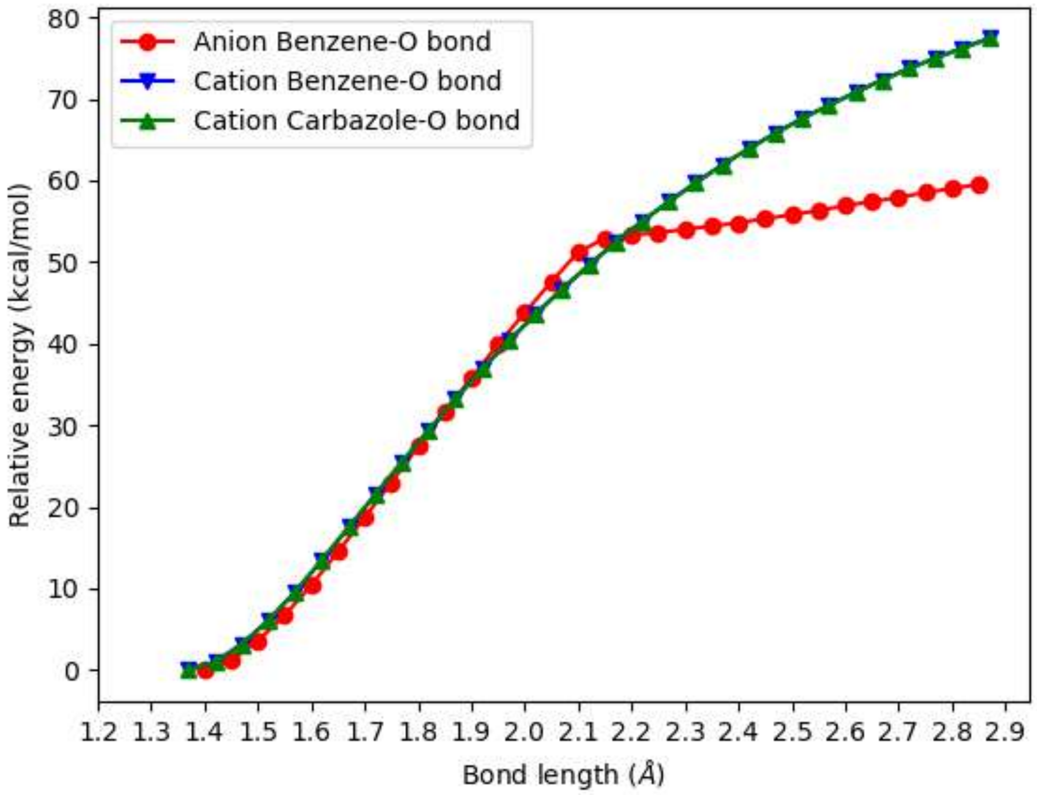

Figure S4. Potential energy surfaces of PtON7-dtb along the elongation of the benzene-O, and Carbazole–O bonds

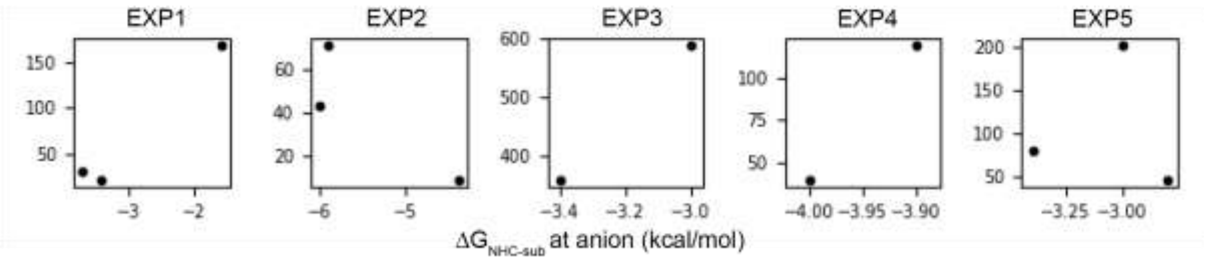

Figure S5. Correlation between $\Delta G_{NHC\text{-}sub}$ of previously reported PtON7-based emitters and their experimental OLED device lifetime. The details of each device are summarized in Table S1. (Units: $\Delta G_{NHC\text{-}sub}$ in kcal/mol; lifetimes in hours)

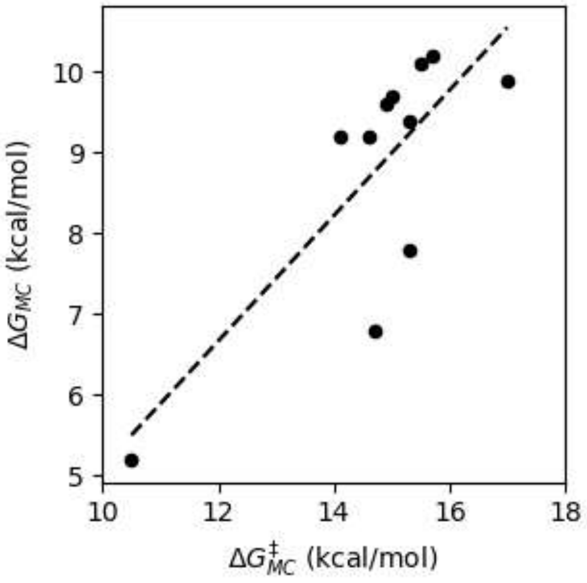

Figure S6. Correlation between $\Delta G^{\ddagger}_{MC}$ and ΔG_{MC} of previously reported PtON7-based emitters. The complete list of emitters is provided in Table S2.

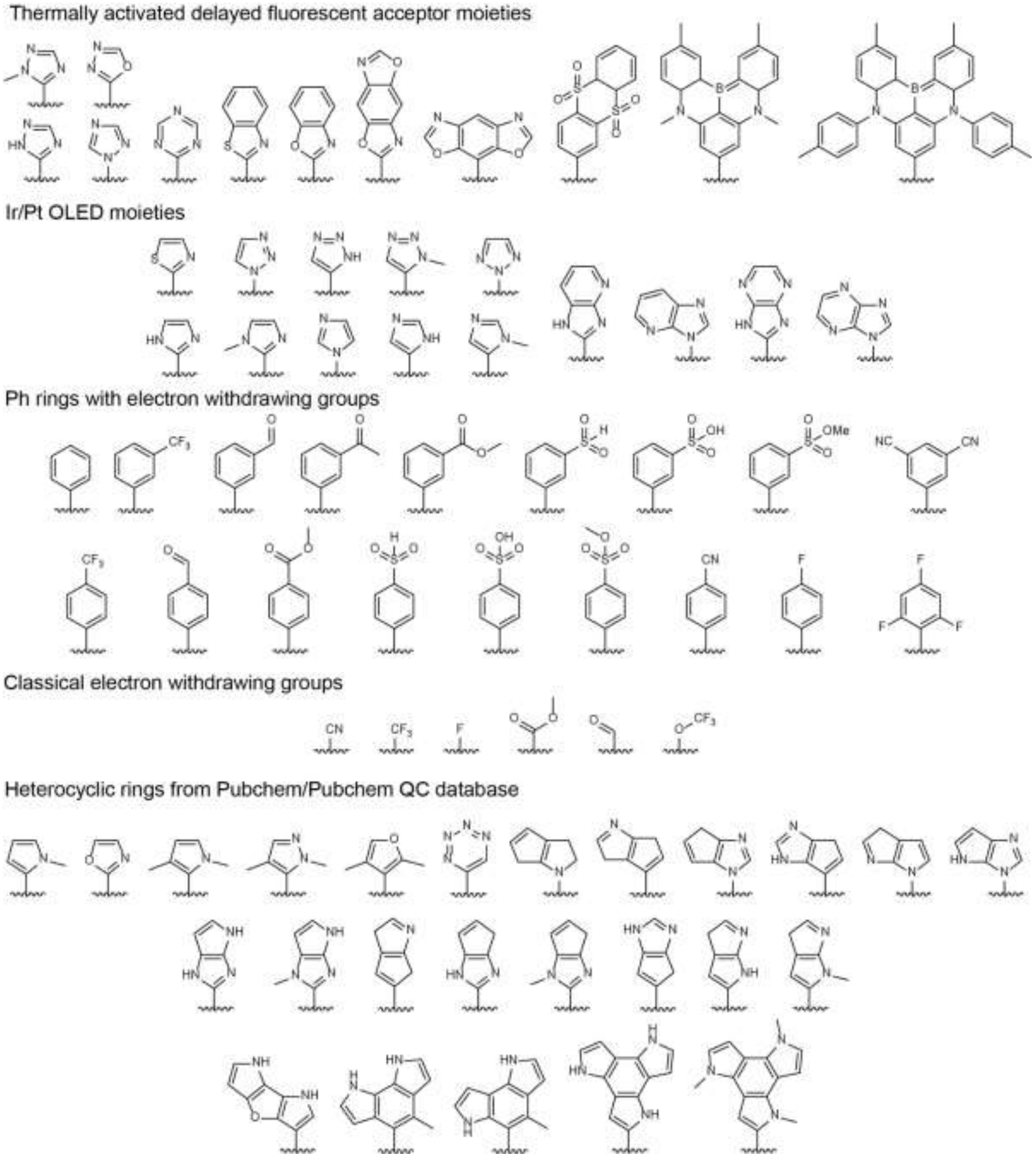

Figure S7. Chemical structures of substituents investigated for the NHC moiety to enhance Pt-pyridine bond stability.

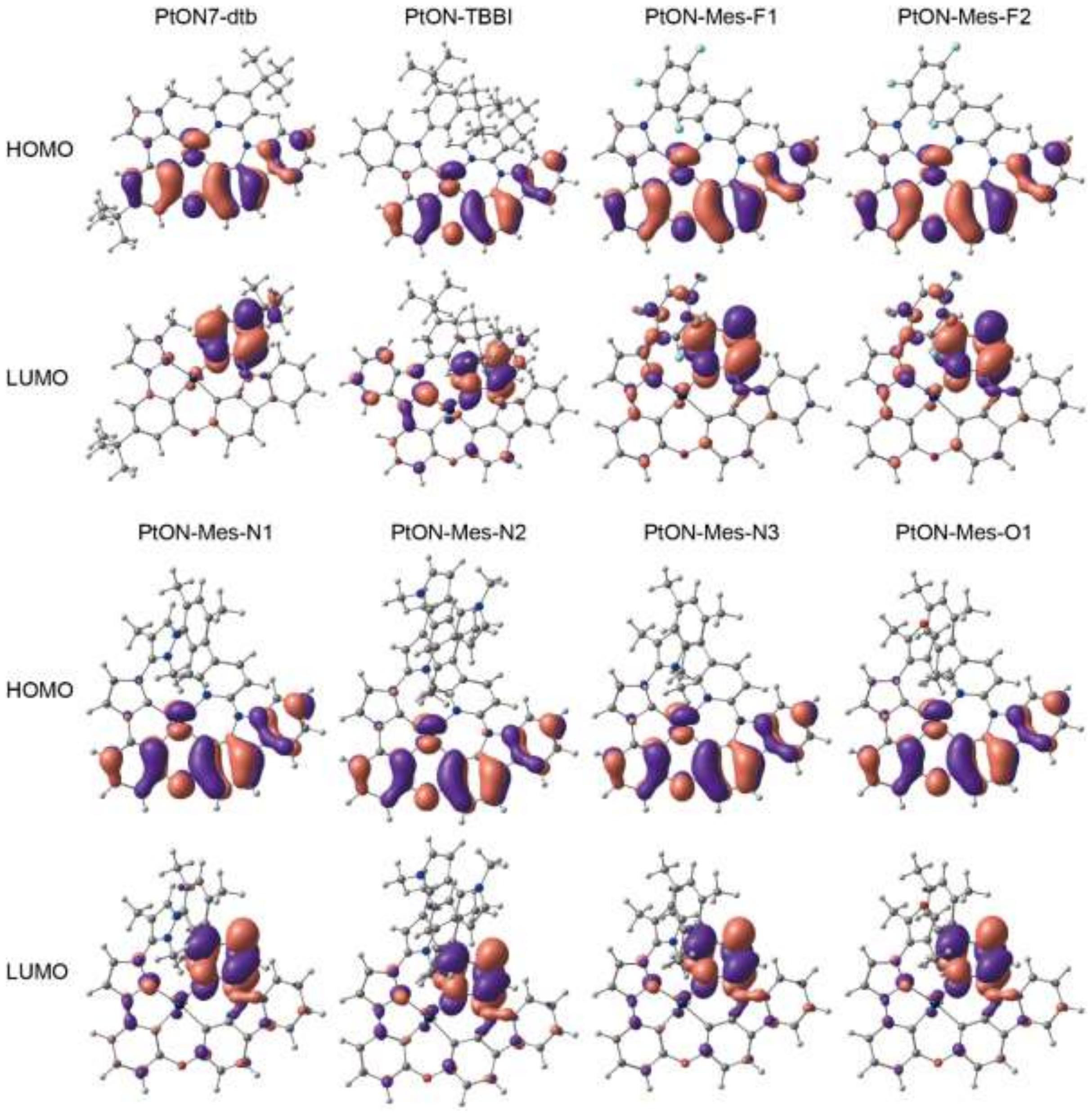

Figure S8. Frontier orbitals of PtON-Mes molecules.

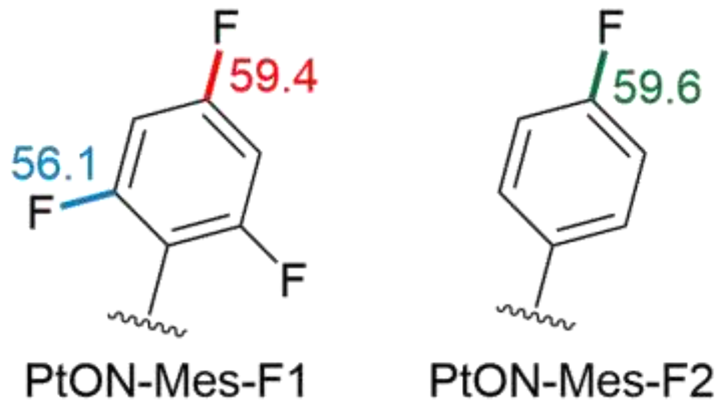

Figure S9. C-F bond dissociation free energies of PtON7-Mes-F1 and PtON7-Mes-F2 in triplet excited state (Unit: kcal/mol)

Table S1. Operational lifetimes and device structures of previously reported Pt-based blue OLED emitters.

	Pt(II) emitters	λ_{max} (nm)	L_0 (cd m^{-2})	Lifetime (hours)	Estimated LT_{95} @L_0=1000nit (hours)†	Device structure	Reference
EXP 1	PtON-TBBI (BD-02)	462	1200	LT_{95} = 20.5	28.5	ITO (50 nm)/HATCN (7 nm)/PCBBiF (45 nm)/ SiCzCz (10 nm)/53 wt.% SiCzCz: 35 wt.% SiTrzCz: 12 wt.% Pt(II) emitters (40 nm)/mSiTrz (5 nm)/mSiTrz: Liq (2:8) (35 nm)/LiF (1.5 nm)/Al (100 nm)	[1]
	PtON-tb-TTB	466		LT_{95} = 31.0	43		
	PtON-tb-DTB	466		LT_{95} = 169.3	235.1		
EXP 2	PtON7-dtb	458	1000	LT_{90} = 8.7	4.4	ITO/HATCN (10 nm)/NPD(60 nm)/SiBCz (5 nm)/10 wt.% Pt(II) emitter:60 wt.% SiBCz:30 wt.%SiTrzCz2 (25 nm)/mSiTrzPh (5 nm)/BPyTP (40 nm)/LiQ/Al	[3]
	PtON5-dtb	464		LT_{90} = 43	21.5		
	PtON5N-dtb	470		LT_{90} = 71	35.5		
EXP 3	PtON-TBBI (BD-02)	460	1000	LT_{70} = 357	59.5	ITO(150 nm)/BCFN:NDP-9 (10nm, 2 wt. %)/BCFN (120nm)/SiCzCz (5nm)/SiCzCz:SiTrzCz2 (40 nm), 10 % of Pt(II) emitters/ miSiTrz (5 nm) miSiTrz:LiQ (10 nm, 50%), BPPB:Yb (10 nm 1 wt. %)/BCFN:NDP-9 (7 nm, 10 wt.%)/BCFN (120 nm)/SiCzCz (5nm)/SiCzCz:SiTrzCz2 (40 nm), 10 % of Pt(II) emitters/ mSiTrz (5 nm)/mSiTrz:LiQ (45 nm, 50 wt.%)/Liq (1.5 nm)/Al (100 nm)	[4]
	Pt-SPCz	460		LT_{70} = 589	98.2		
EXP 4	Pt1N	477	1000	LT_{85} = 40	13.3	ITO (50 nm)/HATCN (10 nm)/BCFN (45 nm)/SiCzCz (5 nm)/SiCzCz:SiTrzCz2 (30nm, 30 %) containing 10 wt.% Pt(II) emitters /mSiTrz (5 nm)/mSiTrz (50 %):Liq (20 nm)/LiF (1.5 nm)/Al (200 nm).	[5]
	Pt2N	478		LT_{85} = 119[c]	39.7		
EXP 5	Pt1	464	1000	LT_{95} = 45.6	45.6	ITO/BCFN: NDP-9 (10 nm)/BCFN (60 nm)/SiCzCz(5 nm)/53 wt.% SiCzCz: 35 wt.% SiTrzCz2: 12 wt.% Pt(II) emitters (35 nm)/mSiTrz (5nm)/mSiTrz: Liq (30 nm)/Yb (1 nm)/Ag (100 nm)	[6]
	PtON-TBBI (BD-02)	459		LT_{95} = 80.5	80.5		
	Pt2	464		LT_{95} = 202	202		
Sun et al., 2022	PtON-TBBI (BD-02)	462	1000	LT_{95} = 150	150	ITO/HAT-CN (10 nm)/BCFN (60 nm)/SiCzCz (5 nm)/SiCzCz:SiTrzCz2:Pt(II) emitter (35 nm, 0.60:0.27:0.13 w/w/w)/mSiTrz (5 nm)/mSiTrz:Liq (31 nm, 5:5)/LiF (1.5 nm)/Al (80 nm)	[7]
Li et al., 2025	Pt2	464	1000	LT_{95} = 290	290	ITO/BCFN: NDP-9 (10 nm)/BCFN (60 nm)/SiCzCz(5 nm)/53 wt.% d-SiCzCz: 35 wt.% d-SiTrzCz2: 12 wt.% Pt(II) emitters (35 nm)/mSiTrz (5nm)/mSiTrz: Liq (30 nm)/Yb (1 nm)/Ag (100 nm)	[6]
	PtON-TBBI	461	1000	LT_{90} = 370	178[b]		[8]

Yuan et al., 2025	PtON-tb-DTB	464	1000	LT_{90} = 557	267[b]	ITO/HATCN (5 nm)/NPB (30 nm)/d-SiCzCz (10 nm)/d-SiCzCz:dSi-TrzCz2:Pt(II) emitters (30 nm, 65:35:10 wt.%)/d-SiTrzCz2 (10 nm)/DPPyA: Liq (40 nm, 50:50 wt.%)/LiF (0.7 nm)/Al (150 nm)	
Zhao, et al., 2025	PtON-TBBI (BD-02)	463	1080	LT_{90} = 215	123.5	ITO (10 nm)/TiN (3 nm)/Ti (3 nm)/Ag (16 nm)/Ti (3 nm)/ITO (40 nm)/BPyTP2 (15 nm)/SiTrzCz2 (5nm)/ SiCzCz:SiTrzCz2:Pt(II) emitter (50nm 43:50:7 vol%)/SiCzCz (5 nm)/NPD (16 nm)/HATCN (12 nm)/BPyTP2: Li (12 nm, 5 vol%)/BPyTP2 (16 nm)/SiTrzCz2 (5 nm)/SiCzCz:SiTrzCz2:Pt(II) emitter (50:43:7 vol%, 50 nm)/SiCzCz (5 nm)/BCFN (5 nm)/HATCN (5 nm)/Ag (120 nm)/Al (3 nm)/Liq (1.5 nm)	[9]

† Lifetimes were standardized to an initial luminance of L_0 = 1000 cd·m^{-2} using an acceleration factor of n = 1.8 [1]. LT_{95} values were obtained via linear interpolation [2].

Table S2. $\Delta G^{‡}_{MC}$, ΔG_{MC}, $\Delta G^{‡}_{NHC\text{-}sub}$, and $\Delta G_{NHC\text{-}sub}$ of previously reported Pt OLED molecules.

Pt OLED molecules	Pt-pyridine bond		NHC-substituent bond		
	$\Delta G^{‡}_{MC}$	ΔG_{MC}	$\Delta G^{‡}_{NHC\text{-}sub}$	$\Delta G_{NHC\text{-}sub}$ at T_1 state	$\Delta G_{NHC\text{-}sub}$ at anion
PtON-TBBI (BD-02)	14.6	9.2	26.7	-2.4	-3.4
PtON-tb-DTB	15.7	10.2	27.0	-0.2	-3.7
PtON-tb-TTB	14.9	9.6	26.3	-2.7	-1.6
Pt-SPCz	15.5	10.1	27.4	-1.7	-3.0
Pt1N	14.1	9.2	26.1	-2.9	-4.0
Pt2N	15.3	9.4	26.3	-2.8	-3.9
Pt1	10.5	5.2	21.9	-7.5	-2.8
Pt2	15.0	9.7	26.0	-2.9	-3.0
PtON7-dtb	14.7	6.8	23.4	-6.9	-4.4
PtON5-dtb	15.3	7.8	23.8	-5.4	-6.0
PtON5N-dtb	17.0	9.9	24.3	-5.0	-5.9

Table S3. Bond dissociation free energies in the T_1 state for the complete detachment of functional groups (R)

moiety / detached functional group	$[Pt\text{-}L^*]^+ + R^-$	$[Pt\text{-}L^*]^- + R^+$	$[Pt\text{-}L^*]^{\cdot} + {\cdot}R$
Pyridine / tert-butyl	192.5	127.4	11.6
Benzene / tert-butyl	187.2	150.4	10.8
N-heterocyclic carbene / methyl	157.1	169.2	-6.9

Table S4. Bond dissociation free energies in the cationic state for the complete detachment of functional groups (R)

moiety / detached functional group	$[Pt\text{-}L^*]^{\cdot} + R^+$	$[Pt\text{-}L^*]^+ + R^{\cdot}$
Pyridine / tert-butyl	92.8	94.6
Benzene / tert-butyl	92.0	89.2
N-heterocyclic carbene / methyl	145.8	66.5

Table S5. Bond dissociation free energies in the anionic state for the complete detachment of functional groups (R)

moiety / detached functional group	$[Pt\text{-}L^*]^{\cdot} + R^-$	$[Pt\text{-}L^*]^- + R^{\cdot}$
Pyridine / tert-butyl	100.8	37.6
Benzene / tert-butyl	100.0	60.5
N-heterocyclic carbene / methyl	87.1	-4.4

Table S6. Energetics of bond dissociation pathways for PtON7-dtb in charged states. (Unit: kcal/mol)

Bond type	Cation		Anion	
	Activation energy	Free energy difference	Activation energy	Free energy difference
Pt-pyridine	23.9	20.0	20.9	6.2
N-heterocyclic carbene - methyl	-[a]	66.5	26.7	-4.4
Benzene - O atom	-[a]	No minimum (Figure S4 blue line)	-[a]	47.9
Carbazole - O atom	-[a]	No minimum (Figure S4 green line)	-[a]	No minimum (Figure S4 red line)
Pt - N-heterocyclic carbene	-[a]	46.0	-[a]	32.1
Carbazole-pyridine	-[a]	43.6	-[a]	51.4[b]
N-heterocyclic carbene -benzene	-[a]	79.7	-[a]	23.6

[a]The transition state optimization was conducted only for Pt-pyridine (cation and anion) and NHC-methyl bonds (anion only), as other dissociation pathways exhibited significantly higher free energy differences than the activation energy of the dissociation of Pt-pyridine bond.
[b]The dissociated pyridine forms a Pt–C bond while the Pt–N bond is cleaved.

Table S7. Electronic structures of PtON7 derivatives according to substituent types and positions on the N-heterocyclic carbene ring.

	S0 state orbital energy			T1 state orbital energy			ΔE_{T1} (eV)	ΔG_{MC} (kcal/mol)
	HOMO (eV)	LUMO (eV)	σ* (eV)	Low energy SOMO (eV)	High energy SOMO (eV)	σ* (eV)		
PtON7 (1-Me)	-4.67	-1.33	0.94	-5.54	-2.06	0.87	2.66	6.6
1-CN	-4.95	-1.55	0.76	-5.28	-2.62	0.57	2.52	12.6
1-CF3	-4.83	-1.32	0.72	-5.44	-2.28	0.72	2.68	10.8
1-NMe2	-4.63	-1.15	1.03	-5.16	-1.97	1.00	2.73	8.6
1-iPr	-4.64	-1.33	0.99	-5.29	-2.02	0.83	2.67	3.7
1-Ph	-4.67	-1.22	1.04	-5.28	-2.05	0.96	2.70	10.3
2-Me	-4.63	-1.3	0.87	-5.25	-2.01	0.88	2.66	6.4
2-CN	-4.96	-1.55	0.68	-5.55	-2.33	0.69	2.68	7.0
2-CF3	-4.85	-1.46	0.76	-5.44	-2.22	0.66	2.68	6.5
2-NMe2	-4.59	-1.27	0.88	-5.20	-1.99	0.93	2.65	6.9
2-iPr	-4.62	-1.3	0.85	-5.25	-2.01	0.88	2.66	5.9
2-Ph	-4.66	-1.32	0.90	-5.27	-2.04	0.94	2.66	6.8
3-Me	-4.64	-1.3	0.96	-5.28	-2.05	0.96	2.66	6.4
3-CN	-4.89	-1.6	0.69	-5.58	-2.44	0.59	2.56	8.4
3-CF3	-4.79	-1.47	0.63	-5.45	-2.26	0.78	2.62	7.0
3-NMe2	-4.43	-1.27	0.98	-5.21	-1.98	0.94	2.65	6.4
3-iPr	-4.63	-1.30	0.95	-5.25	-2.02	0.91	2.65	6.5
3-Ph	-4.62	-1.31	0.95	-5.26	-2.05	0.96	2.64	6.6

Table S8. Electronic structure PtON7 derivatives according to substituent types and positions on the pyridine ring

(Unit: eV)	HOMO energy	LUMO energy	σ^* orbital energy	ΔE_{T1}
PtON7	-4.67	-1.33	0.94	2.66
5-Me	-4.64	-1.30	0.97	2.66
5-tBu	-4.63	-1.24	0.97	2.70
5-Ada	-4.62	-1.25	0.99	2.68
5-PhAda	-4.61	-1.27	1.03	2.67
5-Mes	-4.64	-1.31	0.98	2.64
6-Me	-4.63	-1.21	0.98	2.71
6-tBu	-4.62	-1.22	0.99	2.70
6-Ada	-4.61	-1.18	1.00	2.76
6-PhAda	-4.6	-1.31	1.01	2.66
6-Mes	-4.64	-1.27	0.96	2.62
7-Me	-4.64	-1.32	0.91	2.71
7-tBu	-4.64	-1.31	0.81	2.70
7-Ada	-4.63	-1.28	0.85	2.76
7-PhAda	-4.6	-1.27	0.84	2.66
7-Mes	-4.62	-1.30	0.96	2.62

References

1. Y. H. Jung; G. S. Lee; S. Muruganantham; H. R. Kim; J. H. Oh; J. H. Ham; S. B. Yadav; J. H. Lee; M. Y. Chae; Y. H. Kim; J. H. Kwon. Modified t-butyl in tetradentate platinum (II) complexes enables exceptional lifetime for blue-phosphorescent organic light-emitting diodes. *Nat. Commun.* **2024**, 15, 2977.

2. J.-H. Lee; C.-H. Chen; P.-H. Lee; H.-Y. Lin; M. Leung; T.-L. Chiu; C.-F. Lin. Blue organic light-emitting diodes: current status, challenges, and future outlook. *J. Mater. Chem. C.* **2019**, 7, 5874.

3. G. Li; L. Ameri; B. Dorame; Z.-Q. Zhu; J. Li. Improved Operational Stability of Blue Phosphorescent OLEDs by Functionalizing Phenyl-Carbene Groups of Tetradentate Pt(II) Complexes. *Adv. Funct. Mater.* **2024**, 34, 2405066.

4. H. Lee; B. Park; G. R. Han; M. S. Mun; S. Kang; W. P. Hong; H. Y. Oh; T. Kim. Superbly Efficient and Stable Ultrapure Blue Phosphorescent Organic Light-Emitting Diodes with Tetradentate Pt(II) Complex with Vibration Suppression Effect. *Adv. Mater.* **2024**, 36, 2409394.

5. N. Kim; J. Moon; H. Lee; D. K. You; C. H. Ryu; D. Kim; H. Park; G. Yeom; J. Bin; J. Y. Lee; K. M. Lee. Specific molecular design of tetradentate Platinum(II) complexes for enhancing the electroluminescent performances of blue-phosphorescent organic light-emitting diodes. *Chem. Eng. J.* **2025**, 505, 159169.

6. H. Li; F.-F. Hung; S. Wu; J. Qiu; C. Li; S. Nie; J. Yang; L. Duan; P. Zhou; G. Cheng; C.-M. Che. Deep Blue Tetradentate Pt(II) Emitter Coordinated With Fused luorenyl N-heterocyclic Carbene. High Efficiency, Narrow FWHM, and Superior Operational Lifetime LT95 of 290 h at 1000 cd m^{-2}. *Small.* **2025**, 21, 2409662.

7. J. Sun; H. Ahn; S. Kang; S.-B. Ko, D. Song; H. A. Um; S. Kim; Y. Lee; P. Jeon; S. Hwang; Y. You; C. Chu; S. Kim. Exceptionally stable blue phosphorescent organic light-emitting diodes. *Nat. Photon.* **2022**, 16, 212–218.

8. W. Yuan; T. Huang; J. Zhou; M.-C. Tang; D. Zhang; L. Duan. High-efficiency and long-lifetime deep-blue phosphorescent OLEDs using deuterated exciplex-forming host. *Nat. Commun.* **2025**, 16, 4446.

9. H. Zhao; C. E. Arneson; S. R. Forrest; Stable, deep blue tandem phosphorescent organic light-emitting diode enabled by the double-sided polariton-enhanced Purcell effect. *Nat. Photon.* **2025**, 19, 607–614.
